# Crack Orientation and Depth Estimation in a Low-Pressure Turbine Disc Using a Phased Array Ultrasonic Transducer and an Artificial Neural Network

**DOI:** 10.3390/s130912375

**Published:** 2013-09-13

**Authors:** Xiaoxia Yang, Shili Chen, Shijiu Jin, Wenshuang Chang

**Affiliations:** State Key Laboratory of Precision Measurement Technology and Instrument, Tianjin University, Tianjin 300072, China; E-Mails: shjjin@tju.edu.cn (S.J.); changwenshuang@126.com (W.C.)

**Keywords:** phased array ultrasonic transducer, artificial neural networks, low-pressure turbine disc, crack orientation, crack depth, RBF

## Abstract

Stress corrosion cracks (SCC) in low-pressure steam turbine discs are serious hidden dangers to production safety in the power plants, and knowing the orientation and depth of the initial cracks is essential for the evaluation of the crack growth rate, propagation direction and working life of the turbine disc. In this paper, a method based on phased array ultrasonic transducer and artificial neural network (ANN), is proposed to estimate both the depth and orientation of initial cracks in the turbine discs. Echo signals from cracks with different depths and orientations were collected by a phased array ultrasonic transducer, and the feature vectors were extracted by wavelet packet, fractal technology and peak amplitude methods. The radial basis function (RBF) neural network was investigated and used in this application. The final results demonstrated that the method presented was efficient in crack estimation tasks.

## 1. Introduction

Low-pressure steam turbine discs are critical components in power plants which rotate at high speed throughout the year. With the increase of usage time, stress corrosion cracking may occur in the blade attachment region of the turbine discs, leading to heavy financial losses, and even severe accidents [1–3]. Therefore, the initial crack inspection and the forecast of their propagation are essential to the safe operation of the turbine discs, and reliable methods should be developed to serve in this task. Ultrasonic phased array inspection technology has recently been attracting a great deal of attention in nondestructive evaluation applications [4]. The most desirable feature of phased array inspection is the ability of steering and shaping the sound beam flexibly, which is appropriate for detecting components with complex geometrical shapes. Yang and co-workers obtained crack depth information in the turbine discs from sector images produced by the phased array ultrasonic technique [5]. However, the orientation information of the smaller initial cracks, which is important for the estimation of the crack growth rate, propagation direction and working life of the turbine disc, cannot be distinguished effectively in the sector images. Moreover, the crack orientation can greatly influence the evaluation of crack depth, for the reason that the echo amplitudes vary from reflective surfaces with different directions. As a result, the estimations of the depth and orientation information of the initial cracks are both essential in turbine disc crack detection.

There are some traditional methods for the estimation of flaw size in ultrasonic non-destructive testing. When the flaw size is smaller than the ultrasonic beam diameter, the amplitude-equivalent method is most often used, including the equivalent test specimen method and the AVG curve method; to the contrary, when the flaw size is larger than the ultrasonic beam diameter, the length testing method is always used which includes the 3, 6 and 12 dB methods. However, none of the methods mentioned above take the orientations of the flaws into account, which is an obstacle to the testing of the cracks with different orientations. To solve this problem, crack tip diffraction signals are generally used to detect the location of the crack tip, and then the depth and orientation information can be obtained simultaneously [6]. However, this method has its limitations, that is, the isolation of the crack tip signal requires the face of the crack not to be oriented perpendicular to the direction of beam propagation, and commonly, the early cracks are not big enough to generate tip diffraction signals and cannot be measured in this way. In light of the above, more effective methods are needed to estimate the depths and orientations of smaller cracks in their early stage.

In recent years, artificial neural networks have been proved to be effective for flaw identification and evaluation in ultrasonic non-destructive testing. For instance, in respect of qualitative analysis, neural networks can be used to identify the different flaw types such as cracks, pores and slags in metal welds [7]; for quantitative analysis, neural networks can effectively estimate the crack size [8] and the bonding level of composite materials [9]. Neural networks are nonlinear mapping processes which have significant good self-learning, self-adaptivity, fault tolerance, associative memory capacity and high degree of parallelism features. This kind of model has no special data distribution requirements so that it can efficiently solve non-normal distribution and non-linear problems. Especially for their excellent generalization ability, artificial neural networks have a great advantage and have achieved good application results in various fields. There are many other common pattern recognition methods, such as support vector machine, hidden Markov model, naive Bayes classifier and decision tree which we did not choose in this paper, because they have some disadvantages compared with neural networks in our application. For example, the support vector machine is not suitable for large-scale training data; the hidden Markov model requires prior knowledge of the data statistics; the naive Bayes classifier needs data which must fit a certain statistic distribution; the decision trees may make larger errors as the number of classes increases, and so on. Neural networks also have their own shortcomings, for instance they are prone to plunge into local minima and their training speed is slow, *etc.* Improved neural network models such as the RBF neural network have conquered these problems. This kind of network can process large-scale data, and has simpler structure, faster convergent speed and powerful nonlinearity approach capability in global as well.

In this paper, based on a phased array ultrasonic transducer and artificial neural network, a method to estimate both the depth and orientation of initial cracks in fir-tree type turbine discs, which are the most prevalent in low-pressure turbine rotors, is proposed. In the following sections, firstly, the data collection system was built and the experimental data is described. The A-scan echo signals from cracks with different depths and orientations were collected through a phased array ultrasonic transducer, and the feature vectors were extracted by the wavelet packet, fractal technology and peak amplitude methods. Then, the RBF neural network was investigated for this estimation work. Finally, the estimation results of the neural network were analyzed. Our results showed that the proposed method can efficiently estimate both the depth and orientation of initial cracks in turbine discs.

## 2. Data Collection

**Figure 1.**
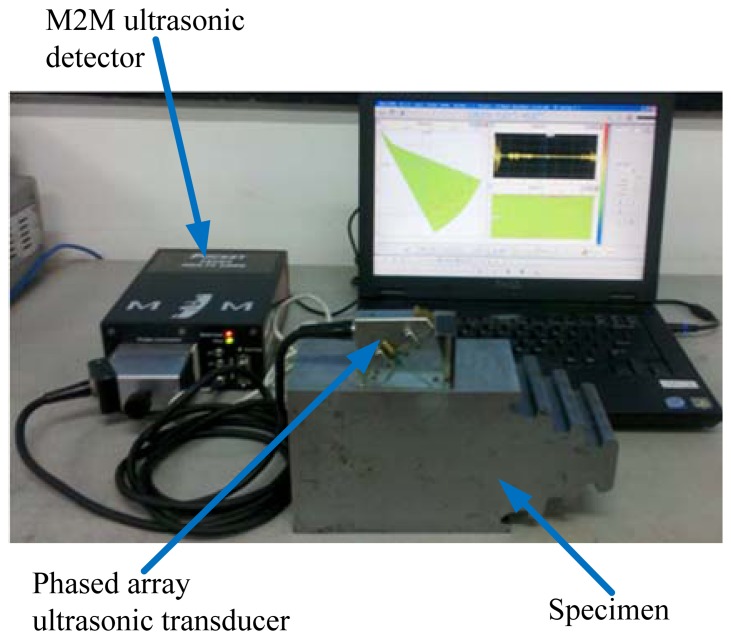
Data collection system.

The experimental data was obtained using the pulse-echo inspection method. The data collection system consists of the following main components: M2M ultrasonic detector, linear phased array ultrasonic transducer with a 36° wedge, the specimen with artificial defects, and a PC. The detector sampling rate is 100 MHz, and a phased array ultrasonic transducer with a center frequency of 5 MHz served as the transmitter and receiver, the number of elements is 64, element width is 0.49 mm, element length is 10 mm and the inter-spacing between centers of adjacent elements is 0.59 mm. For our study, we machined a small part of the turbine disc which is made of steel and has three hooks as the experimental specimen. The whole experimental set up is shown in Figure 1.

**Figure 2.**
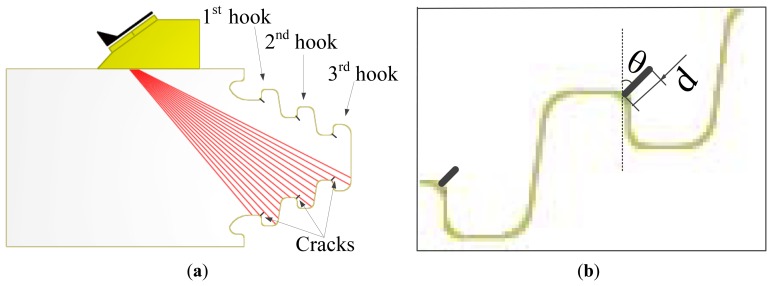
(**a**) Sector scan in the specimen. (**b**) Crack orientation angle and depth.

**Figure 3.**
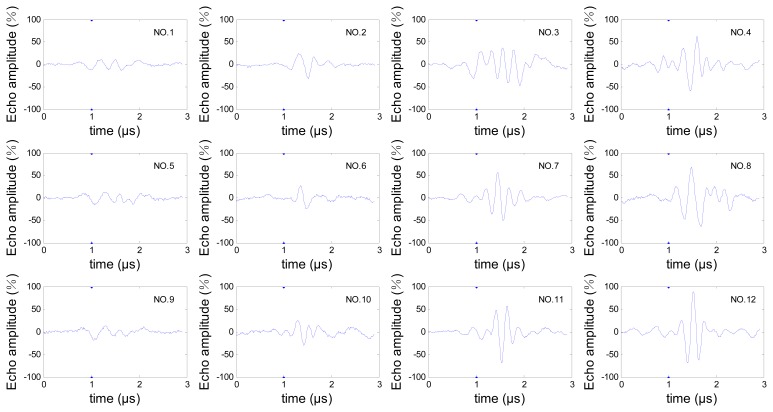
One set of A-scan echo signals from the cracks in the specimen.

**Figure 4.**
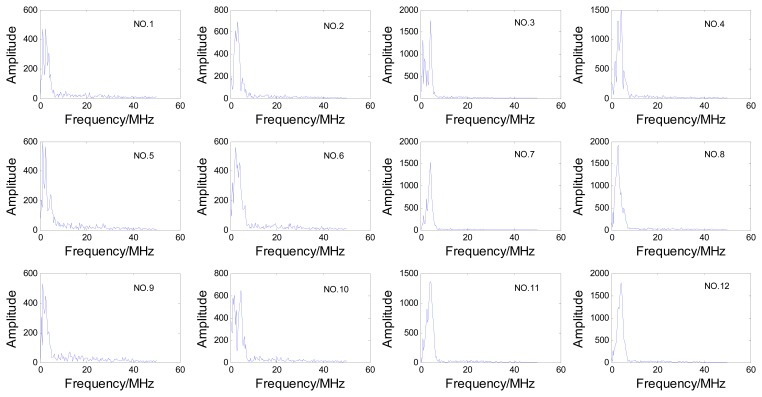
The relevant frequency spectra of the A-scan echo signals.

In order to reduce the transducer movement, for this inspection we chose a sector scan, which can scan all the three hooks without movement in radial direction of the disc, then the A-scan signals from cracks in each hook can be obtained from this sector scan data. The steering beams were not focused because the three hooks are in different depths. The sector scan in the specimen is shown in Figure 2(a), and the orientation angle θ and depth d of cracks are shown in Figure 2(b). We can see that the three hooks are detected by different steering angle beams. To avoid the influence of differences between beams, the estimation works are discussed for the three hooks respectively, so cracks were processed in each hook, which contains depth 0.5 mm, 1.0 mm, 1.5 mm, 2.0 mm and orientation angle 5°, 25°, 45°. Crack depths and orientations are shown in Table 1. All the A-scan signals from these cracks are analyzed for the estimation work. Due to space limitations, only one set of these A-scan signals is shown in Figure 3 and the relevant frequency spectrum is shown in Figure 4 to help us observe the signals preliminarily.

## 3. Feature Extraction

Feature extraction means capturing information that is relevant to different depths and directions of cracks from the ultrasonic echo signals, which is the critical task for the crack estimation. Ultrasonic echo signals always have the characteristics of high level non-linearity, non-stationarity and transient nature. Wavelet and fractal have been proven to be excellent methods for processing this kind of signals. In [10], the wavelet transform is used to analyze lamb-wave ultrasonic NDE signals; in [11], the fractal property of time series serves as a preprocessing tool for the classification of defects probed by ultrasonic signals; in [12], the wavelet transform and fractal theory are combined to analyze the ultrasonic NDE data on the multi-layer adhesive structure. In the following, wavelet packet and fractal analysis were used for feature extraction of echo signals. Furthermore, in the time domain, the echo peak amplitude features were also extracted.

### 3.1. Wavelet Packet Energy Spectrum Extraction

During the detection process of the ultrasonic phased array, the transducers are excited by narrow pulses which are similar to function *δ*, and then the pulsed ultrasound which has extremely short duration time is produced. Therefore, the ultrasound contains such a wealth of frequency spectrum information, that it can be regarded as the superposition of many harmonic components with different frequencies. When ultrasonic waves meet flaws in the specimen, some waves are reflected back. The flaws with different orientations and sizes can be considered as different system functions which can influence amplitude-frequency and phase-frequency characteristics of the incident ultrasonic to various degrees, so signals reflected from different flaws should have considerable differences in frequency band energy.

The wavelet packet is an extension of wavelet analysis which can provide more fineness in signal decomposition than the wavelet. This analysis can decompose the ultrasonic signals into independent frequency bands completely, and then the features of different echo signals can be characterized by the frequency band energy.

#### 3.1.1. A Short Review of Wavelet Analysis

The wavelet transform decomposes a signal into a set of basic functions which are obtained from the mother wavelet. A mother wavelet is a function *ψ*(*t*) which must satisfy the following admissibility condition:

(1) $$\int_{-\infty }^{\infty }\frac{{\left|\hat{\psi }(\omega )\right|}^{2}}{\left|\omega \right|}d\omega <\infty$$

where *ψ̂*(*ω*) is the Fourier transform of *ψ*(*t*). Then the basic functions *ψ_a,b_*(*t*) are generated from the mother wavelet *ψ*(*t*) by dilations and translations:

(2) $$\psi_{a,b}(t)=\frac{1}{\sqrt{\left|a\right|}}\psi (\frac{t-b}{a})$$

where *a*,*b*∈ *R*, *a* ≠ 0 are scaling and shifting factors, respectively. The basic function set *ψ_a_*_,_*_b_*(*t*) are called wavelet family, and the Continue Wavelet Transform (CWT) is defined as followed:

(3) $$CWT_{f}(a,b)=\langle f(t),\psi_{a,b}(t)\rangle =\int_{-\infty }^{\infty }f(t)\psi_{a,b}(t)dt$$

where *f*(*t*) is the signal to be decomposed.

In practice, for the convenience of processing the data by computer, the signals are always a discrete series; therefore, one prefers to write the signal as a discrete superposition of a discrete set of continuous wavelets, which is called Discrete Wavelet Transform (DWT). In the transform, the scaling and shifting factors are discretized and given by *a* = *a*_0_*^j^* and *b* = *kb*_0_*a*_0_*^j^* (*j*,*k* ∈ ℤ. In general, *a*_0_ and *b*_0_ are always set to 2 and 1, respectively. The function family with discretized factors becomes:

(4) $$\psi_{j,k}(t)=2^{-\frac{j}{2}}\psi (2^{-j}t-k)$$

Then the wavelet decomposition can be expressed as:

(5) $$f(t)=\sum_{j,k}d_{j,k}\psi_{j,k}(t)$$

where the *d_j_*_,_*_k_* called wavelet coefficients which are inner products of the signal with the wavelet basis functions:

(6) $$d_{j,k}=\langle f(t),\psi_{j,k}(t)\rangle =\int_{-\infty }^{\infty }f(t)\psi_{j,k}(t)dt$$

Mallat has extensively studied this discrete wavelet transform [13]. He proposed the significant concept of “multiresolution analysis” and built a complete mathematical theory. On the basis of this theory, meanwhile, a fast algorithm for wavelet transforms is presented. It is called Mallat algorithm and has occupied an important position in wavelet analysis.

In the multiresolution analysis, the scaling subspaces *V_j_*(*j* ∈ ℤ to describe the successive approximation spaces are considered. To express the multiresolution analysis, the nested structure of the *V_j_* are shown as: …⊂ *V*_2_⊂ *V*_1_⊂ *V*_0_⊂ *V*_−1_ ⊂*V*_−2_⊂…⊂*L*^2^. The scaling function *ϕ*(*t*) is also introduced, and its dilated and translated versions are expressed as: $\phi_{j,k}(t)=2^{-\frac{j}{2}}\phi (2^{-j}t-k)$, where *j*, *k* ∈ ℤ. Then *ϕ_j,k_*(*t*) constitutes an orthonormal basis of the closed subspace *V_j_*. For each *j*, the *ψ_j,k_*(*t*) spans a wavelet subspace *W_j_* which is exactly the orthogonal complement of *V_j_* in *V_j_*_− 1_, *i.e.*:

(7) $$V_{j-1}=W_{j}⊕V_{j}$$

Then the original signal *f*(*t*) can be decomposed to:

(8) $$f(t)=\sum_{k}c_{j}(k)\phi_{j,k}(t)+\sum_{k}d_{j}(k)\psi_{j,k}(t)$$

where *j* is the level number of the wavelet decomposition, *c_j_* and *d_j_* are the approximation coefficients and detail coefficients of *f*(*t*), respectively.

To construct the mother wavelet *ψ*(*t*), one may first determine the scaling function *ϕ*(*t*) which satisfies the two scale difference equation:

(9) $$\phi (t)=\sqrt{2}\sum_{n}h(n)\phi (2t-n)$$

where *h*(*n*) are low-pass filter coefficients defined by $h(n)=\frac{1}{2}\langle \phi (\frac{t}{2}),\phi (t-n)\rangle$, *n* ∈ ℤ. The mother wavelet *ψ*(*t*) is obtained via the scaling function *ϕ*(*t*):

(10) $$\psi (t)=\sqrt{2}\sum_{n}g(n)\phi (2t-n)$$

where *g*(*h*) = (−1)*^n^h*(1 − *n*) which are high-pass filter coefficients and orthogonal to *h*(*n*).

Then the Mallat algorithm for the computation of the decomposition coefficients can be summarized by the following equations:

(11) $$c_{j}(k)=\sum_{m}h(m-2k)c_{j-1}(m)$$

(12) $$d_{j}(k)=\sum_{m}g(m-2k)c_{j-1}(m)$$

where *m* ∈ ℤ.

The reconstruction of *c_j_*_− 1_(*k*) can be expressed as:

(13) $$c_{j-1}(k)=\sum_{m}c_{j}(m)h(k-2m)+\sum_{m}d_{j}(m)g(k-2m)$$

#### 3.1.2. Wavelet Packets

The wavelet packet method is a generalization of wavelet decomposition that can provide more sophisticated analysis. Wavelet analysis decomposes signals into two parts: low-frequency and high-frequency. During the course of decomposition, only the low-frequency part is decomposed into two parts and this decomposition can be continued to a number of deeper levels. It can be seen that, in the wavelet decomposition, the frequency resolution reduces in higher frequency. Wavelet packet transform is more accurate in signal decomposition, as it decomposes signals not only in the low-frequency part, but also in the high-frequency part.

For the convenience of discussion, in wavelet packet analysis, scaling subspace *V_j_* and wavelet subspace *W_j_* are expressed by a uniform subspace $U_{j}^{n}$:

(14) $$\begin{array}{l} U_{j}^{0}=V_{j}\\ U_{j}^{1}=W_{j},j\in \mathbb{Z} \end{array}$$

Then the formula (7) can be rewritten as:

(15) $$U_{j-1}^{0}=U_{j}^{0}⊕U_{j}^{1},j\in \mathbb{Z}$$

and the recursion formula can be expressed as:

(16) $$U_{j-1}^{n}=U_{j}^{2n}⊕U_{j}^{2n+1},j\in \mathbb{Z},n\in {\mathbb{Z}}^{+}$$

Define the subspace $U_{j}^{n}$ is the closure space of function *u_n_*(*t*). Making *u*_0_(*t*) = *ϕ*(*t*) and *u*_1_(*t*) = *ψ*(*t*), the function of wavelet packet is made up as follows:

(17) $$u_{2n}(t)=\sqrt{2}\sum_{k}h(k)u_{n}(2t-k)$$

(18) $$u_{2n+1}(t)=\sqrt{2}\sum_{k}g(k)u_{n}(2t-k)$$

where *g*(*k*) = (−1)*^k^h*(1 − *k*) which is high-pass filter and orthogonal to the low-pass filter *h*(*k*).

Set $f_{j}^{n}(t)\in U_{j}^{n}$, $d_{j}^{2n}$ and $d_{j}^{2n+1}$ are the wavelet packet coefficients of subspaces $U_{j}^{2n}$ and $U_{j}^{2n+1}$, respectively. The fast decomposition and reconstruction algorithms of wavelet packet can be obtained led by the Mallat algorithm:

(1) Wavelet packet decomposition algorithm is described as:
  (19) $$d_{j}^{2n}(k)=\sum_{l}h(l-2k)d_{j-1}^{n}(l)$$
  (20) $$d_{j}^{2n+1}(k)=\sum_{l}g(l-2k)d_{j-1}^{n}(l)$$
  where *l* ∈ ℤ.
(2) Wavelet packet reconstruction algorithm is described as:
  (21) $$d_{j-1}^{n}(k)=\sum_{l}h(k-2l)d_{j}^{2n}(l)+\sum_{l}g(k-2l)d_{j}^{2n+1}(l)$$

#### 3.1.3. Wavelet Packet Energy Spectrum

**Figure 5.**
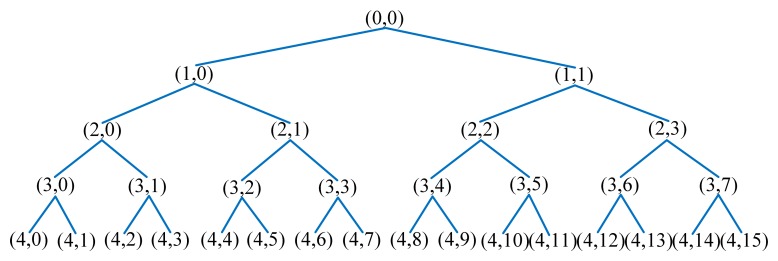
Wavelet packet decomposition tree of four levels.

Energy spectrum feature extraction by wavelet packet can be described as four steps:

(1) Decompose the ultrasonic echo signal.
  We decompose the ultrasonic echo signals into four layers, and the decomposition coefficients can be acquired from low frequency to high frequency of 16 sub-bands in the fourth layer. The wavelet packet decomposition tree of four levels is shown in Figure 5, in which (*j*,*i*) represents the *i^th^* node of the *j^th^* level. The wavelet packet coefficients of these frequency components in each level from low frequency to high frequency are represented by *X_j_*_0_, *X_j_*_1_, *X_j_*_2_ … *X*_*j*(2^*j*^−1)_ (*j*=0, 1, 2…).
(2) Reconstruct decomposition coefficients.
  Reconstruct the wavelet packet decomposition coefficients, and extract the frequency range signal *S*_4_*_i_* (*i* = 0, 1, 2…15). Then the total signal *S* is the sum of each frequency band signal:
  (22) $$S=S_{40}+S_{41}+S_{42}\ldots +S_{4(15)}$$
(3) Calculate energy of each frequency band.
  The energy of signal *S*_4_*_i_* can be calculated as:
  (23) $$E_{4i}=\int {\left|S_{i}(t)\right|}^{2}dt=\sum_{k=1}^{N}{\left|x_{4ik}\right|}^{2}(k=0,1,2\ldots N)$$
  where *N* is the number of discrete points of signal *S*_4_*_i_*, and *x*_4_*_ik_* is the amplitude of the *k^th^* discrete point.
(4) Construct feature vectors.
  The energy spectrum feature vector of the 4*th* level can be constructed as follows:
  (24) $$T_{4}=[E_{40},E_{41},E_{42}\ldots E_{4(15)}]$$

In order to accommodate the analyze model in the following section, the energy spectrum vectors are normalized as follows:

(25) $$T_{4}^{'}=[E_{40}/E_{4},E_{41}/E_{4},E_{42}/E_{4}\ldots E_{4(15)}/E_{4}]$$

where $E_{4}=\sum_{i=0}^{15}E_{4i}$.

**Figure 6.**
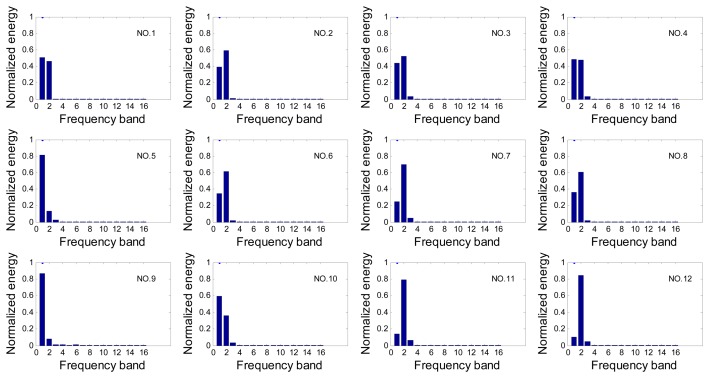
Wavelet packet energy spectrum of one set of echo signals.

To illustrate this abstract feature extraction method, the wavelet packet energy spectrum of one set of data is shown in Figure 6 as an example.

In the level 4, the main frequency bands which concentrate most energy of the crack echo signal, *i.e.*, the first three bands of $T_{4}^{'}$, constitute feature vector *T*:

(26) $$T=[T_{4}^{'}(1),T_{4}^{'}(2),T_{4}^{'}(3)]$$

### 3.2. Fractal Feature Extraction

Fractal theory has been developed into a powerful tool in dealing with non-linear problems in natural science and engineering. In nonlinear signal processing, fractal dimensions can be used to quantitatively analyze the signal irregularity and complexity. The combination of wavelet and fractal is proposed according to the unity that the multi-scale decompositions and self-similarity in both wavelet transform and fractal theory possess, that is, the ability to analyze the signal information from low-resolution to high-resolution of the wavelet transform is consistent with the fractal method which gets more and more abundant details through transforming the signal from big scale to small scale.

In the fractal analysis, the more tortuous, convoluted and richer in detail, the higher the fractal dimensions. The box-counting dimension is one of the best known fractal dimensions which can be easily defined and obtained numerically. In this study, fractal box-counting dimensions are combined with wavelet packet transform and extracted as features for crack echoes.

#### 3.2.1. Fractal Theory

Fractal theory was introduced by Mandelbrot [14] and is associated with the geometrical properties of an object. At first, this theory is used to study set topology, and it was later applied to natural science and engineering. A fractal is a kind of mathematical abstraction used to describe the regularity of many irregular things and phenomena. It has revealed the unification between confirmation and randomicity, order and disorder in non-linear systems. So far fractals have not been given a strict mathematical definition yet, but the basic properties of the fractal object can be summarized by the following two points: firstly, fractal objects possess the characteristic of self-similarity, which has described by the fact that fractal objects have similar features in the local as well as global sense. That is to say, some quantitative properties will not change with enlarging or shrinking operations. Secondly, the irregularity and complexity degree of the fractal object can be reflected by the fractal dimensions. The fractal dimension is a quantitative index used to describe the self-similarity level which is a break from the traditional definition of dimension. In this study, box-counting dimensions were calculated and used to extract features for crack echoes.

#### 3.2.2. Calculation of Box Dimension

The box dimension of a set *S* contained in n-dimension Euclidean space *R^n^* is defined as follows:

(27) $$Dim_{B}S=\underset{\delta \to 0}{\lim}\frac{Lg\;N_{\delta }\;(S)}{-Lg\;\delta }$$

where *S* is arbitrary non-empty bounded subset of *R^n^*, *δ* is the side-length of meshes, and for any *δ* > 0, *N_δ_*(*S*) is the minimum number of meshes needed to cover *S*.

In actual fact, the box dimensions of the discrete time domain signals cannot be obtained under the condition of *δ* → 0, because the highest resolution of discrete time series is the sampling interval Δ, so the approximate method is usually used to compute it in practice, *i.e.*, taking *l*Δ (*l* = 1, 2, 3…L, L < *N*) as the side-length of meshes, where *N* is the number of discrete points of signals. The minimum number of meshes in side length of *l*Δ that cover a discrete signal *S* is noted as *N_l_*_Δ_(*S*) and can be calculated as:

(28) $$N_{l\Delta }(S)=\sum_{m=1}^{N/l}\text{ceil}\left\{\left|\max(S[l(m-1)+1],S[l(m-1)+2],\ldots ,S[l(m-1)+l+1])-\min(S[l(m-1)+1],S[l(m-1)+2],\ldots ,S[l(m-1)+l+1])\right|/(l\Delta )\right\}$$

where ceil( ) means taking the upward integer.

According to formula (27), we can obtain *Dim_B_*(*S*), *i.e.*, the box-counting dimension of discrete signal *S* as:

(29) $$\mathrm{lg}N_{l\Delta }(S)=-Dim_{B}(S)•\mathrm{lg}(l\Delta )+C$$

where *C* is a constant. It is shown that −*Dim_B_*(*S*) is the slope of the straight line in the *N_l_*_Δ_(*S*)−lg(*l*Δ) graph which is fitted by the least square method. The start point of the zone is noted as *l_s_* and the end point is noted as *l_e_*, and (*l_s_*Δ, *l_e_*Δ) is named the scale-free interval.

So, the box dimension estimating method for the discrete time domain ultrasonic signals can be summarized as follows:

(1) Generate the meshes. According to the approximate method, take *l*Δ (*l* = 1, 2, 3…L, L < *N*) as the side-length of meshes, where Δ is the sampling interval and *N* is the number of discrete points of the signals.
(2) Cover the signal with boxes whose side-length are *l*Δ (*l* = 1, 2, 3…L, L < *N*), and calculate the corresponding minimum number of meshes *N_l_*_Δ_(*S*) that cover signal *S*.
(3) Plot *N_l_*_Δ_(*S*)−lg(*l*Δ) In the scale-free interval, the curve is a straight line which is fitted by least square method, and the box dimension *Dim_B_*(*S*) is given by the slope of this straight line.

In our present study, box-counting dimensions were calculated for the frequency band signals extracted in Section 3.1., *i.e.*, the signals *S*_4_*_i_* shown in formula (22). In order to accommodate the analyze model in the following section, the box-counting dimensions are subtracted by 1, and the fractal feature vector is constituted as:

(30) $$D=[Dim_{B}(S_{41})-1,Dim_{B}(S_{42})-1,Dim_{B}(S_{43})-1]$$

To show the identifiability of this impalpable feature, the fractal features of one set of crack signals are shown in Table 2 as examples.

### 3.3. Echo Amplitude Feature Extraction

**Figure 7.**
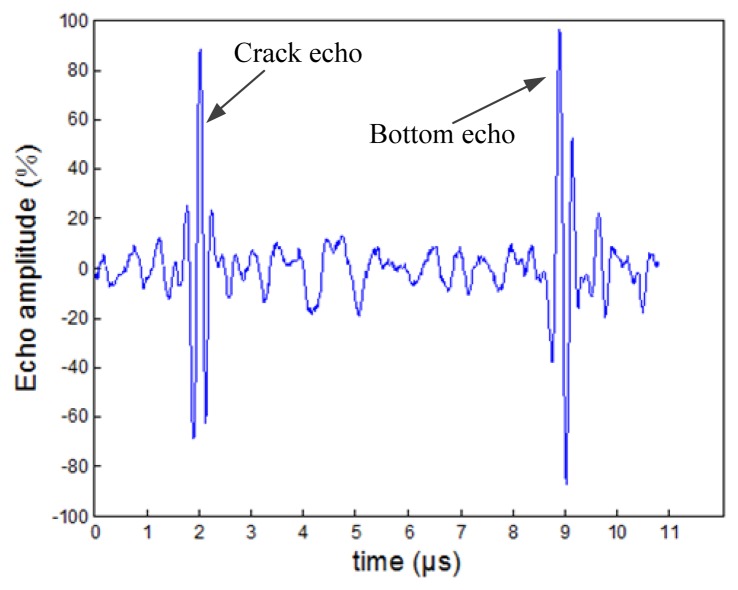
Crack echo wave and bottom echo wave.

Peak amplitude of an ultrasonic echo signal is a typical feature which is easily interpreted visually and widely used in quantitative detections. Besides the frequently-used flaw echo amplitude, echo amplitude from bottom under the flaw is also an effective characteristic parameter. The bottom echo can assist to perceive larger flaws which have weak echos because of the influence of geometry, reflectivity or the reflective surface direction, so this feature should be considered in the present issue. The crack echo wave and bottom wave are shown in Figure 7. In this part, the peak amplitudes of crack echoes noted *P* and the peak amplitude ratio of crack echo to bottom echo noted *R* are both extracted as the amplitude features in time domain. The echo amplitude feature vector can be shown as:

(31) A=[P,R]

### 3.4. Feature Vector Construction

Up to now, a total of eight features are extracted by wavelet packet, fractal and echo amplitude, then the feature vector F can be constructed as:

(32) F=[T,D,A]

This 8-dimensional vector will be the input of the estimation model proposed in the next section.

## 4. RBF Neural Network

In this work, the RBF neural network is used for the estimation. The RBF neural network is an important supervised learning tool of machinery learning technology which can perform arbitrary nonlinear mapping from the input space *R^d^* to the output space *R^n^* with arbitrary accuracy. This model has faster processing speed and global approximation, is free from the local minima problem.

**Figure 8.**
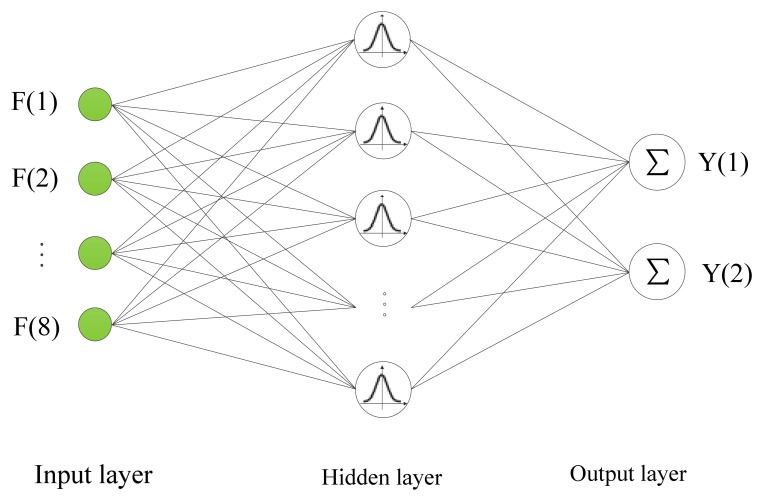
Structure of the RBF neural network.

The RBF neural network is a multi-input, multi-output forward networks model which has three layers consisting of an input layer, a hidden layer, and an output layer. In this work, the 8-dimensional feature vector F constructed in Section 3 is the input of the neural network, and the crack orientation angle and depth constitute the 2-dimensional output, then the structure of the RBF neural network is shown in Figure 8 [15].

The input layer sends the input variables to each neuron in the hidden layer. The activation function applied to neuron in hidden layer is radial basis function. In RBF neural network, the Gaussian function is the most common radial basis function, so the activation function of the *i^th^* neuron in the hidden layer can be expressed as:

(33) $$R_{i}=\exp(-\frac{{\left\|F-c_{i}\right\|}^{2}}{2\sigma_{i}^{2}})(i=1,2\ldots m)$$

where *m* is the number of neurons in the hidden layer, *F* is the input vector, *c_i_* and *σ_i_*, respectively, are the center and the width (or spread) of the Gaussian function of the *i^th^* neuron in the hidden layer, and ‖*F* − *c_i_*‖ represents the distance between *F* and *c_i_*.

The output layer is a linear combination of the hidden layer output with associative weights and biases, and the *j^th^* output of the RBF network can be calculated as:

(34) $$Y_{j}=\sum_{i=1}^{m}w_{ij}R_{i}-b_{j}\;(j=1,2\ldots n)$$

where *n* is the number of neurons in the output layer, *w_ij_* is the weight between *i^th^* neuron in the hidden layer and *j^th^* neuron in the output layer, *b_j_* is bias of the *j^th^* neuron in the output layer.

## 5. Estimation Results and Analysis

**Figure 9.**
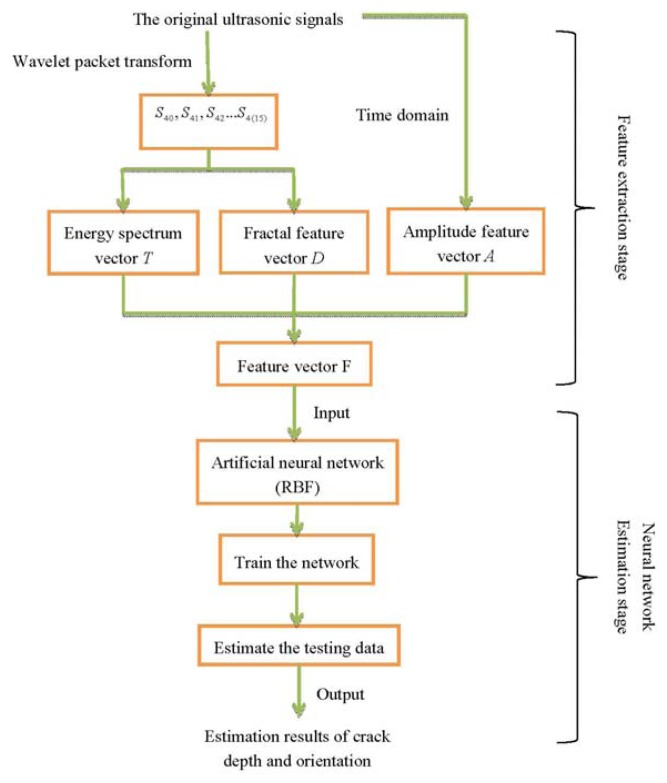
The flowchart of the presented estimate method.

**Figure 10.**
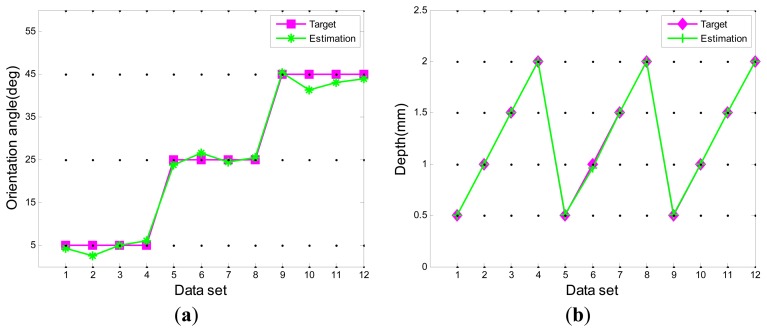
The test results of the model. (**a**) Crack orientation angle; (**b**) Crack depth.

The presented estimate method can be summarized by the flowchart illustrated in Figure 9:

In this section, we take the crack echo signals in the 2nd hook as an example to analyze the performance of the proposed model. The 240 available data samples were divided into 80% for training and 20% for testing the networks. The partial test results of the model for estimation of the crack orientation angle and depth are given in Figure 10.

The estimation results show that the proposed method can evaluate the crack orientation angle and depth with a reasonable level of accuracy. Especially for the depth estimation, fairly small errors occur. Orientation angle estimation results show greater errors than the depth estimation, but the error level is still within an acceptable range. In order to analyze the errors accurately, the root mean square errors (RMSE) of the testing data are calculated as:

(35) $$\text{RMES}=\sqrt{\frac{\sum {\left(Y_{i,t}-Y_{i,e}\right)}^{2}}{n}}$$

where *Y_i_*_,_*_t_* is the testing data, *Y_i_*_,_*_e_* is the corresponding estimation data, and *n* is the number of testing data. The calculation results show that the RMSE of the testing data are 1.613 and 0.012 for the crack orientation angle and depth, respectively. The results indicate that the proposed method had a good performance in the estimation of crack orientation angle and depth in turbine discs.

## 6. Conclusions

Stress corrosion cracks in low-pressure steam turbine discs are serious hidden dangers for production safety in power plants, and the initial crack inspection and the forecast of their propagation are essential to the safe operation of the turbine discs. In order to estimate the crack orientation and depth at an early stage, a method based on a phased array ultrasonic transducer and an artificial neural network was proposed in our study. The A-scan echo signals from cracks with different depths and orientations were collected in the lab using a phased array ultrasonic transducer, and the feature vectors were extracted by the wavelet packet, fractal technology and peak amplitude methods. Then, a RBF neural network was investigated for this estimation work and the estimation results were analyzed. The test results showed that the proposed model was a useful tool to estimate both the depth and orientation of initial cracks in turbine discs. At the present stage, all the research is still in the laboratory and the experimental data is collected from the machined specimen which is not enough in practice. In our future work, a field data acquisition system will be developed and the field data will be collected and analyzed using our model.

## Figures and Tables

**Table 1.** Orientation and depth of crack in the specimen.

**NO.**	**Orientation (deg)**	**Depth (mm)**
1	5	0.5
2	5	1.0
3	5	1.5
4	5	2.0
5	25	0.5
6	25	1.0
7	25	1.5
8	25	2.0
9	45	0.5
10	45	1.0
11	45	1.5
12	45	2.0

**Table 2.** The fractal features of one set of crack signals.

	**NO.1**	**NO.2**	**NO.3**	**NO.4**	**NO.5**	**NO.6**	**NO.7**	**NO.8**	**NO.9**	**NO.10**	**NO.11**	**NO.12**
Band 1	0.167	0.184	0.165	0.175	0.164	0.178	0.165	0.205	0.155	0.167	0.247	0.191
Band 2	0.245	0.217	0.246	0.280	0.314	0.206	0.236	0.246	0.325	0.261	0.237	0.220
Band 3	0.395	0.382	0.354	0.396	0.418	0.348	0.295	0.382	0.404	0.331	0.302	0.311

## References

[1] Gray J.L. (1972). Investigation into the consequences of the failure of a turbine-generator at Hinkley Point ‘A’ power station. Proc. Inst. Mech. Eng..

[2] Mukhopadhyay N.K., Chowdhury S.G., Das G., Chattoraj I., Das S.K., Bhattacharya D.K. (1998). An investigation of the failure of low pressure steam turbine blades. Eng. Fail. Anal..

[3] Morita A., Kagawa H., Sugawara M., Kondo Y., Kubo S. (2006). Evaluation of corrosion fatigue crack propagation life at low-pressure steam turbine rotor groove. Eng. Fract. Mech..

[4] Zhong Z.M., Mei D.S. (2002). Development and application of ultrasonic phased array technique. (In Chinese). Nondestruct. Test..

[5] Yang S., Yoon B., Kim Y. (2009). Using phased array ultrasonic technique for the inspection of straddle mount-type low-pressure turbine disc. NDT E Int..

[6] Mak D.K. (1985). Ultrasonic methods for measuring crack location, crack height and crack angle. Ultrasonics.

[7] Yi W., Yun I.S. (1998). The defect detection and non-destructive evaluation in weld zone of austenitic stainless steel 304 using neural network ultrasonic wave. KSME Int. J..

[8] Zhang Y.H., Wang L.H., Zhu H.L. Ultrasonic Crack Size Estimation Based on Wavelet Neural Networks.

[9] Li L., Zhou R., Xu H. Application of Self-Organizing Neural Network in Ultrasonic Detection of Faults in Bonding Composite Material.

[10] Legendre S., Massicotte D., Goyette J., Bose T.K. (2000). Wavelet-transform-based method of analysis for lamb-wave ultrasonic NDE signals. IEEE Trans. Instrum. Meas..

[11] Vieira A.P., de Moura E.P., Goncalves L.L., Rebello J.M.A. (2008). Characterization of welding defects by fractal analysis of ultrasonic signals. Chaos Solitons Fractals.

[12] Dun Y., Chen J.H., Wang G.L., Shi X.H., Xu Z.S. Identification of Multilayered Structure Properties Using Wavelet-Fractal Dimension of Ultrasonic Data.

[13] Mallat S.G. (1989). A theory for multiresolution signal decomposition: the wavelet representation. IEEE Trans. Pattern Anal. Mach. Intell..

[14] Mandelbrot B.B., Wheeler J.A. (1983). The fractal geometry of nature. Amer. J. Phys..

[15] Zeng S.W., Hu H.G., Xu L.H., Li G.H. (2012). Nonlinear adaptive PID control for greenhouse environment based on RBF network. Sensors.

